# Avian Stress-Related Transcriptome and Selenotranscriptome: Role during Exposure to Heavy Metals and Heat Stress

**DOI:** 10.3390/antiox8070216

**Published:** 2019-07-10

**Authors:** Isidoros Seremelis, Georgios P. Danezis, Athanasios C. Pappas, Evangelos Zoidis, Kostas Fegeros

**Affiliations:** 1 Chemistry Laboratory, Department of Food Science and Human Nutrition, Agricultural University of Athens, 75 Iera Odos, 11855 Athens, Greece; 2 Department of Nutritional Physiology and Feeding, Faculty of Animal Science, Agricultural University of Athens, 75 Iera Odos, 11855 Athens, Greece

**Keywords:** Animal health, heat stress, heavy metals, selenium, selenoprotein, selenotranscriptome

## Abstract

Selenium, through incorporation into selenoproteins, is one of the key elements of the antioxidant system. Over the past few years there has been increased interest in exploring those molecular mechanisms in chicken, responsible for the development of this protection system. In more detail, Cd/Pb poisoning and heat stress increase oxidation, mRNA levels of inflammatory proteins, and apoptotic proteins. Selenium seems to enhance the antioxidant status and alleviates these effects via upregulation of antioxidant proteins and other molecular effects. In this review, we analyze avian transcriptome key elements with particular emphasis on interactions with heavy metals and on relation to heat stress.

## 1. Introduction 

Selenium (Se) is characterized as a vital nutrient for animals and humans [1,2,3]. In the form of selenocystein (Sec), Se is integrated into selenoproteins to participate in various organism and cellular functions [4,5]. The main characteristic of all selenoproteins, is the presence of Sec in their peptides [6,7,8]. A stem-loop structure named selenocysteine insertion sequence (SECIS) is responsible for the decoding of a stop codon UGA to Sec (U) [9]. In mammalian genomes, integration of Se in selenoprotein requires the existence of SECIS. On the other hand, dietary Se deficiency, in diverse species, has been implicated in various diseases. In chicken, Se deficiency (SD) is characterized by the appearance of pendulous ventral part of the neck region giving a soft feel on palpation [10]. Also, SD results in loss of appetite, swollen legs, uncoordinated movement, poor feathering, and poor growth [11]. The Se shortage symptoms may appear in broiler chicks utilizing unsupplemented diets [12,13,14]. 

Cadmium (Cd) is a heavy metal and extremely toxic. It is accumulated into birds’ organs mainly through the feed and on elevated concentrations can cause acute or chronic poisoning [15]. One of Cd toxicity mechanisms is the nitric oxide (NO) overproduction and the expression of inducible NO synthase (iNOS) which governs NO synthesis. In splenic lymphocytes of chicks, exposure to Cd elevates the activity of NO and iNOS. Cadmium harms the liver, kidney, nerves, bones, and other organs [16]. In chicken, Cd exposure can cause altered behavioral responses and decreased egg production [17]. Specifically, it has been found that Cd could cause autophagy in chicken pancreas and alter the concentration of trace elements in kidney [18,19,20]. Additionally, the overproduction of NO affects *Bcl-2, p53* and other genes, stimulating the release of cytochrome c (Cyt-c) and thereafter apoptosis [21]. As in the chicken, kidney of rabbits is the target organ of Cd toxicity. In rabbits, Cd is accumulated in various organs such as liver, kidney, and lung, impairing the functions of these organs [22,23]. Furthermore, reduction of cell antioxidant capacity and cell apoptosis is induced by Cd poisoning [24,25]. Also, exposure to Cd could lead in renal dysfunction [26]. It has been showed that Cd, in sublethal concentration, has notably changed the levels of glycogen in kidneys of silver carp (*Hypophthalmichthys molitrix*) and triggered kidney damage [27,28]. 

Lead (Pb) is another extremely toxic heavy metal that is broadly distributed in nature due to human activities. Lead can seep into the environment via various ways, including sewage discharges and burning of fossil fuels [29]. It has been reported, that vegetables may contaminate with Pb through burning of municipal waste and by emissions from industry. In leafy vegetables the accumulation of Pb, is directly related to atmospheric Pb through absorption from leaves [30,31]. It is recognized, that Pb has toxic effects on several organs and systems in whole organism including central nervous system, blood system, heart, and kidney [32]. Furthermore, Pb exposure targets immune organs. Lead can impact the humoral immune function of organisms. It decreases the host’s resistance to several pathogen infections. According to duration of Pb exposure and dose, it may be capable of reducing serum immunoglobulin levels [33]. It seemed that T lymphocytes are the most sensitive to the hazardous effects of Pb. Further, Pb interferes with the TH1/TH2 lymphocytes balance [34], causing immune dysfunction triggered by inflammation [28]. Additionally, Xing et al. showed that Pb alters heat shock proteins (HSP) and cytokines mRNA expression and thus decreased immune function in chicken neutrophils [35]. Similar effects of Pb have been reported in several other avian species [36]. In detail, Gasparik et al. reported that Pb in pheasants is accumulated in kidneys, livers, pectoral muscles, eggs and ovaries, and reduced egg hatching rate, fertilization rate, and egg weight [37]. Also, Butkauskas and Sruiga showed that Pb was hazardous for fertility, hatchability, and reproductive success of Japanese quails [38]. Lead poisoning also affects heat shock proteins expression. Huang et al. demonstrated that Pb poisoning raises mRNA expressions of *HSP27, HSP40, HSP60, HSP70*, and *HSP90* in the chicken testes. However, mRNA expression of *HSP40* was the lowest in the Pb group and it was found about 24 times higher compared to the control group. These outcomes indicate that Pb toxicity results in higher mRNA expressions of HSPs [36]. 

Heavy metals are extremely poisonous for avian species. They cause dysfunctions in many organs and alter expression levels of many inflammation factors and antioxidative agents such as selenoproteins. In the later part of this review, we discuss in detail the effects of Cd/Pb toxicity on avian transcriptome and the benefit of Se supplementation.

## 2. Avian Transcriptome Response to Cadmium Toxicity and the Benefits of Selenium Supplementation 

### 2.1. Inflammation Transcripts, Apoptotic Factors and Selenotranscriptome

The predominant mediators of inflammation, cytokines, play a critical role in the inflammatory response induced by Cd exposure and other various environmental challenges in living organisms. Some studies have demonstrated that immune cells inflammatory response is closely related to the expression of inflammatory cytokines. TNF-α is the most important cytokine, activating neutrophils and lymphocytes, promoting the synthesis and release of other cytokines [39]. On the other hand, as a pro-inflammatory factor, the main function of iNOS in inflammatory neutrophils is the induction of NO production. Nitric oxide is synthesized under inflammatory conditions and takes part in immunoregulation [40,41]. Li-li Liu et al. demonstrated that Cd exposure of broiler cerebrum significantly raised *iNOS* mRNA levels, Cd accumulation and NO production. Furthermore, Cd induced brain damage by adjusting iNOS–NO system changes [42].

It has been reported that Cd stimulation causes the expression of ICAM-1 via NF-κB activation in cerebrovascular endothelial cells similar to findings by Liu et al. [42]. Moreover, Låg M et al. have shown that the mRNA expression levels of IL-1β and TNF-α were reduced after exposure to Cd in lung cells of rats [43]. Furthermore, in another study, exposure to Cd increased mRNA expression of *IL-1B* while decreased *IL-17, IL-10, IL-4, IL-2,* and *IFN-γ* in chicken splenic lymphocytes indicating an impact on immune responses. Supplementation with Se decreased Cd toxicity and in addition the mRNA expression levels of *IL-17, IL-10, IL-2, IL-4,* and *IFN-γ* were higher than in the Cd alone treated group. However, the mRNA expression levels were not as high as in the Se alone treated group and the control group. Expression levels of mRNA of *IL-2* were also higher in Cd and Se treated group than in Cd group. Additionally, in respect to the control group, the mRNA levels of *IL-10* and *IL-4* in chicken splenic lymphocytes were decreased dramatically due to Cd toxicity [44].

Moreover, Cd exposure could trigger immune cells to raise the mRNA expression of *IL-10* and *IL-1B*. At gene level, the altered gene expression regulation of cytokines induced the raise of IL-4 and suppression of IFN-γ production. Cadmium could influence the immune inflammation of neutrophils by modifying the regulation of cytokine expression as it turned out via the suppression of IL-17 production, the raise of IL-4 release and the production of proinflammatory cytokines IL-1B, TNR-α, IFN-γ, and IL-10. Also, Cd moderately induced the pro-inflammatory cytokines NF-κB, iNOS, TNF-α, COX-2, and PGE2. Furthermore, inflammation caused by Cd raised iNOS activity and NO production. Analogous results were demonstrated in the levels of NF-κB protein expression [45,46].

The key step for enabling downstream caspases, a family of protease enzymes playing essential roles in programmed cell death and inflammation, is the activation of Bak, a member of the proapoptotic Bcl-2 family, at the mitochondria. This has a crucial role in the regulation of apoptosis under chronic endoplasmatic reticulum stress (ERS). The role of ERS in inflammation and stress is not unilateral, ERS can be triggered by a variety of inflammatory factors, and many inflammatory diseases are associated with ERS. Similarly to previous reports on Cd toxicity and ERS, Chen et al. showed that Cd induces high expression of TNF-α, IL-1b, IL-4, and IL-10 in peripheral blood neutrophils of broiler, acting as strong inducer of ERS and activating ER pathways, one of which is ATF6 cleavage [46]. When Cd triggers ERS, the apoptosis pathway is conducted by the ATF6 branch. Higher apoptotic cells populations were found in the Cd group compared to the control group. Also, the mRNA expression of caspase-12 in the Cd-induced chicken peripheral blood neutrophils model group was significantly higher than that in the control group. The results in the protein expression levels of caspase-12 also illustrates that it is an important molecule, which initiates apoptosis selectively in response to ERS [46]. Furthermore, Chen et al. proposed that in the lack of apoptosis, the mRNA levels of caspase-9, *GRP78*, and caspase-3 increased significantly while levels of *CaM* and *Bcl-xL* decreased in the peripheral blood neutrophils of chicken in the Cd treatment group [46].

On the other hand, in hens liver, Cd toxicity caused significant increase of *IL-1β, TNF-α, COX-2, PTGES,* and *NF-κB* mRNA levels while similarly, in hens serum, *TNF-α,* and *IL-1b* were significantly increased [47]. Recently, a study by Chen et al. showed that Cd exposure caused deregulation of miRNA-33-AMPK axis, further suppressed AKT/mTOR and HSP70-NF-κB/JNK signaling pathway and triggered BNIP3-dependent autophagy in chicken spleen [48]. Moreover, another study showed that Cd toxicity increased mRNA expression of caspase-3, *Cyt-c*, caspase-9, *Bax, p53*, and protein levels of caspase-3, Bax, and Cyt-c in liver of chicken. However, Cd toxicity reduced Bcl-2 protein and mRNA levels. These findings pointed that Cd induced apoptosis in chicken hepatocytes by the NO-mediated mitochondrial-dependent pathway and that Cd-triggered apoptosis was mediated by the pro-apoptotic genes *Bax, p53,* and *Bcl-2* [45]. 

Messenger-RNA expression of inflammatory cytokines plays a crucial role in inflammatory response. Under inflammatory conditions due to Cd exposure, NO levels and inflammatory cytokines are increased in avian tissues. Also, Cd toxicity induces strongly ERS. In addition, Cd toxicity induces apoptosis on chicken liver and neutrophils via *Bak* activation and triggers BNIP3-dependent autophagy on chicken neutrophils. Some ways on how Se supplementation may alleviate Cd toxicity are proposed.

### 2.2. Selenium Supplementation 

There is little information about possible interactions of Se and Cd in mRNA expression of inflammation factors and selenoprotein genes. A study on Se/Cd treated chicks showed that Se co-administration with Cd alleviated the increase of *COX-2, NF-κB, PTGES,* and *TNF-α* mRNA levels caused by Cd in chicken kidneys. The mRNA levels of *NF-κB* and *TNF-α* slightly increased in Se/Cd treated group in relation to control group but *PTGES* and *COX-2* mRNA levels were not influenced [28]. Cadmium toxicity also induced energy metabolism disorders and mitochondrial damage and Se supplementation reduced mRNA expression of autophagy-related genes (*Atg5, LC3-I, LC3-II, Beclin 1, dynein*) caused by Cd treatment [49].

Also apoptosis and the mRNA level of *Bak, p53*, caspase-3, caspase-9, and *Cyt-c* increased significantly, and *Bcl-2, Bcl-xl,* and *CaM* decreased in chicken splenic lymphocytes of Cd treatment groups. Furthermore, Amantana et al. showed that Cd exposure decreased induction of the promoter of selenoprotein W in rat myoblast and glial cells, which indicates that Cd targets some selenoproteins [50]. Cadmium decreased the expression of selenoprotein S, selenoprotein T, selenoprotein N, and selenoprotein K which are located in the endoplasmic reticulum. Moreover, the protection of Se may be related to the regulation of selenoproteins in chicken lymphocytes [51]. Selenoprotein W has essential role in redox regulation during induction of Ca^2+^ leakage in muscles [52]. As a result, selenoproteins may play major role in Cd toxicity process as well as in Se antagonistic function and are an important link between Cd and Se. These studies indicated that Cd treatment influenced the expression of selenoprotein genes, which may be targets of Cd toxicity. They show correlations between most of selenoproteins. Hence these selenoproteins function similarly in Se antagonism of Cd [53]. It was shown that Cd can reduce the expression levels of selenoprotein N, -T, -K, and -S resided in ER and that Se protection property may be related to the regulation of selenoproteins [51]. On the other hand, another study on Cd exposure and Se co-administration showed that the levels of mRNA of *GPX1* and *TRXR1* were obviously lower in the Cd group compared with other groups. However, the Cd/Se co-treatment significantly attenuated this decrease. Selenium supplementation alone with Cd treatment significantly increased the mRNA of *GPX1* and *TRXR1*, similar to the immunoblotting results of GPX1 and TRXR1 [54].

In a study examining Cd exposure and chicken’s kidney selenotranscritome regulation, there was a significant difference between the control group and Se-treated group in the mRNA levels of all 25 selenoprotein genes (selenoprotein T, selenoprotein N, selenoprotein W, selenoprotein K, Selenoprotein U, selenoprotein S, selenoprotein O, selenoprotein M, selenoprotein I, selenoprotein H, selenoprotein 15, selenoprotein Pb, *SPS2, Sepp1, Sepx1, DIO3, DIO2, DIO1, GPX4, GPX3, GPX2, GPX1, TXNRD3, TXNRD2,* and *TXNRD1*) [28]. Among the control group and Cd-treated group there was a crucial difference of mRNA of selenoprotein W, selenoprotein U, selenoprotein T, selenoprotein S, selenoprotein Pb, selenoprotein O, selenoprotein N, selenoprotein K, *DIO3, GPX3,* and *GPX2* levels, but not in the mRNA expression levels of another 14 selenoprotein genes (selenoprotein 15, selenoprotein M, selenoprotein I, selenoprotein H, *Sepp1, Sepx1, SPS2, DIO2, DIO1, GPX4, GPX1*). In the group with Se/Cd treatment, the mRNA expression levels of selenoprotein W, selenoprotein U, selenoprotein T, *GPX3,* and *GPX2* alleviated, while the amount of alleviation was minor than that in the group with Cd treatment. The expression levels of selenoprotein N, selenoprotein K, selenoprotein S, selenoprotein O, selenoprotein M, selenoprotein I, selenoprotein H, selenoprotein 15, selenoprotein Pb*, Sepx1, Sepp1, SPS2, DIO3, DIO2, DIO1, TXNRD3, TXNRD2, TXNRD1, GPX4,* and *GPX1* were not affected [28,55]. Additionally, treatment with Se through the reduction of MDA levels and the increase of Se-dependent antioxidant enzymes activities in chicken kidney tissues protected them from Cd toxicity [28,56].

Apart from the studies mentioned above, Se protection mechanism against Cd toxicity is based on antioxidant proteins. Also, in another study, Se ameliorated Cd-induced oxidative stress through regulation of mRNA levels of *GPX4* and selenoprotein P [57]. However, Se supplementation, decreased mRNA levels of proapoptotic proteins caspase-3, caspase-9, p53, *Bax* and *Cyt-c*. Moreover, mRNA levels of *Bcl-2* raised when concurrently supplemented Se and Cd in chicken nutrition [45].

## 3. Avian Transcriptome Response to Lead Toxicity and the Benefits of Selenium Supplementation

### Inflammatory Response

Under chemical or noxious physical stimuli, one of the most important indicators of tissue damage is inflammation. The NF-κB signaling pathway has a significant role in inflammation regulation via the transcription of diverse target genes, including iNOS, COX-2, and TNF-α. Regarding COX-2 and iNOS, they are significant enzymes that mediate processes of inflammation [58]. iNOS influences the lung inflammatory response through regulation of chemokine synthesis [59]. Moreover, TNF-α is known as a pro-inflammatory cytokine which may participate in the nonalcoholic fatty liver disease initiation [60]. Activation of NF-κB within endothelial cells constitutes a critical step in the rheumatoid arthritis pathogenesis of experimental models [61]. Pro-inflammatory response can be increased by Pb. Furthermore, Pb decreases cell function in macrophages, possibly by enhancing TNF-α release and oxidative damage [62]. Cadmium-induced inflammatory reaction was weakened by Se, which was interceded, at least in part, by COX-2, iNOS, and TNF-α expression down-regulation, through the NF-κB activation suppression. Therefore, a defensive role of Se is indicated regarding Cd-induced inflammatory response [42].

Selenium could as well antagonize Pb-induced damage on inflammatory cytokines gene expression in chickens’ peripheral blood lymphocytes [63]. Chicken testes is the target organ where Pb accumulates and induces inflammation injury [64]. Also, Pb induced apoptosis in chicken testes. Jiao et al. reported that Pb toxicity raised MDA content; decreased GSH content; and decreased SOD, GPX and GST activities in chicken bursa of Fabricius [65]. Huang et al. noted that Pb poisoning increased mRNA expression of *ATF4, PERK*, caspase-3, caspase-12, *eIF2α,* and *CHOP* in chicken testes [66]. This indicated that Pb induces apoptosis via CHOP/caspase-3 signal pathway and ER stress in chicken testes. The activation of a major ER stress transducer, the dissociated PERK, activates PERK which leads to the elF2α phosphorylation and in sensitization of transcription factor ATF4. Upregulation of CHOP is caused by sustained ATF4 overexpression. Furthermore, CHOP is crucial mediator of ER stress-induced apoptosis. Under standard conditions, CHOP is expressed at low rates and under extended ER stress, CHOP is expressed at high levels and triggers apoptosis. After ER stress, caspase-12 dissociates and is activated and further activates caspase-3. Then, caspase-3 performs apoptosis.

Wang et al. showed that Se supplementation alleviates accumulation of Pb in testes of chicken [67]. Additionally, other studies demonstrated that Se alleviates Pb-induced oxidative stress. Likewise, Huang et al. illustrated that SOD, GPX, and GST activities were reduced by Se co-administration with Pb in chicken testes. Also, Se supplementation had protective effects on apoptosis. Jin et al. showed that triggered apoptosis and caspace-3 raised by Pb poisoning on chicken kidneys was alleviated through Se supplementation [68]. Additionally, Wang et al. showed that Pb poisoning triggers mRNA expression increase of caspace-3, caspace-12, *ATF4,* and *GRP78,* and alleviation occurs via Se supplementation in chicken kidneys [69].

In the same way, Pb-induced increase of *CHOP*, *eIF2α,* and *PERK* mRNA expression and these effects were alleviated by Se in chicken testes [36]. These results indicate that Pb-induced apoptosis, oxidative stress, and ER stress are alleviated by Se via CHOP/caspase-3 signal pathway in the chicken testes [36]. Futhermore, Li et al. in a study on Pb-Se interaction found that Se supplementation alleviated the activation of NF-kB pathway through the reduction of *NF-κB, COX-2* and *TNF-α* expression in chicken neutrophils [70]. 

Lead, in addition, induces the mRNA expression of *NF-κB, TNF-α, COX-2, PTGEs, iNOS, HSP27, HSP40, HSP60, HSP70*, and *HSP90*, the NO content, and the iNOS activity in chicken liver. Also in this case, Se supplementation alleviated those changes [31]. Likewise, mRNA expression of *iNOS, NF-κB, COX-2,* and *TNF-α*, NO content and Pb deposition was ameliorated via Se supplementation in the testes of chicken [67]. In the same way, the results of another study indicated that Se alleviated the changes caused by Pb exposure on Bcl-2 mRNA and protein expression, the increased NO content, the iNOS activity, the relative mRNA and the protein expression of iNOS, the ER-related genes, and caspase-3 and caspase-12 protein expression. Selenium attenuated those changes caused by Pb and Pb-induced apoptosis via ER stress in chicken kidneys. In agreement with the above, Zhao et al. demonstrated that Se supplementation alleviated Pb induced apoptosis, PI3K/Akt pathway suppression and oxidative stress in chicken splenic lymphocytes [71].

In chicken neutrophils the mRNA expression levels of 23 selenoproteins (except of GPX3) and TNF-α, NF-κB, and iNOS were dramatically raised during Pb poisoning. However, Se supplementation alleviated this trend of inflammation factors, increased the mRNA levels of selenoproteins and decreased serum Pb content. Li et al. found positive correlations among inflammatory factors, besides COX-2. COX-2 might have played a principal function in Se antagonism against Pb [70]. Jiao et al. demonstrated that in chicken bursa of Fabricious, Se co-supplementation with Pb alleviated the inflammatory factors (*IL-4, IL-6, IL-12β*, and *IL-17*) mRNA levels increase that were caused due to Pb poisoning [65]. In accordance with previous studies, Xing et al. demonstrated that Pb treatment increased significantly the expression of *TGF-β4, IL-4, IL-8, IL-10, IL-12, IL-1β, IL-1R* and reduced that of *IFN-γ* and *IL-2*. Neutrophil injury and impair immune function in chicken could be induced by Pb poisoning [35]. There was a correlation between five HSPs and nine cytokines. IFN-γ and IL-2 were negatively correlated with other parameters, but there was a positive correlation between *TGF-β4, IL-12, IL-10, IL-8, IL-4, IL-1R, IL-1β, HSP90, HSP70, HSP60, HSP40,* and *HSP27*. In contrast, *HSP70, HSP60,* and *HSP40* were highly related to *IL-1β* [35]. Zhu et al. [72] showed that Pb triggered apoptosis in chicken embryonic neurocytes and brain tissues via mitochondrial pathway. In brain tissues of chicken, Pb induced a time-dependent effect on the decrease of selenoprotein M, *GPX4*, and in embryonic neurocytes Pb induced the mRNA decrease of selenoprotein U. Furthermore, multivariate correlation analysis demonstrated positive correlations between twenty-five selenoproteins; four apoptosis-related genes (caspase-3, *Cyt-c, p53,* and *Bax*); and between *Bcl-2* and the selenoproteins in the embryonic neurocytes and chicks brain tissues [72]. 

In chicken hearts, mRNA expression levels of *PTGEs, COX-2, TNF-α,* and *NF-κB* were decreased with implementation of Se supplementary diet compared with the Pb diet group. The results indicated that Se antagonized Pb induced inflammation [73]. Also, Se protects from Pb poisoning and alleviates the decrease of mRNA levels of *SPS2, Sepx1*, selenoprotein 15, selenoprotein M, *Sepw1, TXNRD1, TXNRD3, TXNRD3, DIO1, DIO3*, *Sepn1*, selenoprotein K, selenoprotein S, selenoprotein T, selenoprotein H, selenoprotein I, selenoprotein U, selenoprotein Pb, *Sepp1*, selenoprotein O, *GPX2, GPX3,* and *GPX4* [73]. Similarly, another study showed that during Pb toxicity the mRNA levels of *NF-κB, TNF-α, COX-2*, and *iNOS* in chickens’ peripheral blood lymphocytes were significantly higher than in the control [63]. In correspondence with previous research outcomes, the results proposed that excess Pb could cause inflammation of chicken peripheral blood lymphocytes. However, Se supplementation decreased Pb toxicity-induced increase of HSP (27, 40, 60, 70, 90), *COX-2, TNF-α, iNOS, HO-1,* and *NF-κB* [63]. Gao et al. [74] , illustrated that dietary Se ameliorated Pb toxicity in the cartilage tissue of broiler chicken. More specifically, Se alleviated the downtrend of the expression of selenoprotein *GPX4, GPX2, GPX1, DIO1, DIO2, TXNRD2, TXNRD3, Sepx1*, selenoprotein O, selenoprotein K, selenoprotein M, selenoprotein T, selenoprotein W, *Sepn1*, selenoprotein 15, selenoprotein I, and selenoprotein U triggered by Pb exposure in the meniscus cartilage. Additionally, in the sword cartilage, Se alleviated the downtrend of mRNA expression of *DIO2, DIO3, TXNRD1, TXNRD2*, selenoprotein K, selenoprotein W, selenoprotein I, selenoprotein H, *SPS2, Sepx1*, selenoprotein 15, selenoprotein O, selenoprotein M, selenoprotein P, selenoprotein T, selenoprotein n1, *GPX2, GPX3,* and *GPX4* induced by Pb toxicity [74]. 

Finally, 25 selenoprotein genes (*TXNRD1, TXNRD2,* and *TXNRD3*, selenoprotein H, selenoprotein I, selenoprotein K, selenoprotein T, selenoprotein M, selenoprotein W, selenoprotein Pb, selenoprotein S, selenoprotein O, selenoprotein U, *DIO1, DIO2, DIO3,* selenoprotein 15, *Sepn1, Sepp1, Sepx1, SPS2, GPX1, GPX2, GPX3,* and *GPX4*) in chicken testes showed highest expression levels in the Se supplemented groups than in the Pb exposed chicken groups indicating an increase of the antioxidative potential due to Se supplementation. Also, there were positive correlations between the selenoproteins gene expression and the expression of five HSPs (*HSP40, HSP27, HSP90, HSP60* and *HSP70*) [36]. 

From the aforementioned, it can be concluded that Se supplementation can alleviate the toxic effects of Pb, by increasing the antioxidative potential in several chicken tissues via upregulation of many antioxidant proteins. A summary of selected studies in chicken describing the effects of heavy metals on various factors, HSP, and selenoproteins is shown in Table 1.

## 4. Heat Stress and Avian Transcriptome 

### 4.1. An Overview of Heat Stress

Regarding heat stress (HS), the poultry industry is sensitive to economic losses due to poor thermotolerance of broiler chickens [77,78]. St-Pierre et al. estimated that the livestock industry experiences a total loss of $1.7 billion (USD) per year due to the negative effects of heat stress [77]. Heat stress has adverse impacts on a variety of performance parameters such as reduced meat quality, egg production, and feed intake of broilers [79,80]. Moreover, the above physiological imbalances of broilers, during heat stress, lead to hormonal disequilibriums [81,82,83,84] as well as decreased immune [85] and reproductive development [84].

Heat stress causes reduction of body weight gain and feed consumption [86], and is therefore a major concern for the poultry industry. Moreover, HS disrupts barrier function and affects enteric development [87]. Heat stress raises radiant heat loss through the redistribution of blood flow from the core body to the periphery. It increases also mortality and feed conversion efficiency, thus lowers carcass weight and lastly decreases broiler meat quality [88]. Moreover, it causes depletion in mineral (Se, Fe and Zn) and vitamin (A and E) tissue concentrations [89] that might be a consequence of the impaired intestinal absorption, due to leakage of the gut. The decreased Se, Zn, and Fe levels outcomes in impairment of oxidative capacity [90]. The changes triggered by HS increase reactive oxygen species (ROS) formation [91,92,93] and interrupt the balance of antioxidant defense system and oxidation. This triggers the occurrence of lipid peroxidation and oxidative damages to biochemical molecules such as proteins and DNA [91].

Heat stress has been shown to lead to diminished growth rate in various studies [94]. Adomako et al. [95] noted that HS prompt to higher protein degradation, while both Gu et al. [96] and Li et al. [97] exhibited that HS leads to apoptosis as well. Caspase-6 constitutes a part of a gene family of caspases related to the apoptotic processes. It was observed that twelve days post-HS, CASP6 mRNA expression levels were raised, as part of a cascade developed by animals under HS to limit the accumulation of proteins. Additionally, HS results in ROS augmentation, and it seems that a concomitant raise in ROS concur with CASP6 expression to promote apoptosis [98,99]. When broilers were exposed to HS, blood and nutrient flow to gastrointestinal tract were reduced, causing ATP depletion, intestinal hypoxia, intracellular acidosis as well as nitrosative and oxidative stress together with altered intestinal integrity and function [100]. Lipopolysaccharide leakage was raised due to intestinal permeability, leading to multiple failure of organs [100]. 

Furthermore, meat quality was degraded under HS conditions. Poultry have increased contents of polyunsaturated fatty acids in muscles and are extremely sensitive to oxidative stress when exposed to high temperatures [101,102]. Postmortem glycolysis also rose due to oxidative stress caused by HS. Additionally, meat quality of broiler was impaired because of tissue glycogen conversion into lactic acid (protein and texture contents) [88,103,104].

Slawinska et al. [105] showed that HS environment induced iNOS activity upregulation in a chicken macrophage-like cell line. On another study [106], in contrast with common conditions, due to HS, NO content and iNOS activity were increased in the spleen of broilers. This indicated that under HS conditions, the antioxidant defense system is disrupted leading to ROS accumulation and in subsequent release of a big number of intermediaries of inflammation. Yao’s research [107] demonstrated that ER stress response may be downstream from oxidative stress. Xu et al. [108] showed that under HS environment, in chicks spleen, *GRP94* and *GRP78* mRNA levels considerable raised which clearly indicates that ER stress occurs by HS. Also, multiple molecular pathways such as ATF6, PERK, and IRE1 have interrelation with stress in ER. During ER stress, the expression of the above genes were elevated [106]. In the next chapter, we examine in more detail the way that HS alters the avian transcriptome.

### 4.2. Avian Transcriptome Response to Heat Stress 

Heat stress alters the mRNA expression of oxidants thereby increasing cellular ROS. The NOX family is consisted of seven members (DUOX1 and -2, NOX1, -2, -3, -4, and -5] [109]. Upon the outcomes of the study by Habashy et al., some NOX enzymes encoding genes were upregulated due to long term heat stress in broilers [110]. Bánfi et al.’s [111] results demonstrated that NOX activator 1 and NOX organizer 1 (NOXO1) activate NOX1, the enzyme that generates superoxides. For instance, subunit p22phox was essential for the activation of NOX3 [112]. Although, in lack of activators, NOX3 superoxide production was raised via NOXO1 and PHOX organizers [113]. An N-terminal domain is contained in DUOX2 (like peroxidases) and DUOX2 produces hydrogen peroxide [114]. Three canonical EF-hands (calcium binding domains) are contained in N terminus of NOX5 and this differentiates it from other NADPH oxidases. In addition, superoxide is generated by NOX5 in response to intracellular Ca^2+^ [115]. 

Under Ca^2+^ activation, NOX5 is activated and produces high superoxide amounts and further exhibits another function by becoming a proton channel for charge compensation and pH changes as a result of the electron export [116]. Regarding HUVEC cells exposed to HS, cytoplasmic Ca^2+^ peaked 1 h after HS and later reduced progressively. Further, at 1 h, a primary raise concerning mitochondrial Ca^2+^ was noted, which peaked at 9 h and reduced at 12 h post-HS [96]. Calcium level in chickens’ plasma was increased 2 h post-HS but reduced to control levels, 24 h post-HS. In this way, it would be expected that NOX5 will be down-regulated 24 h post-HS as was noticed in that study. At 1 and 12 days post-HS, NOX2 was down-regulated. Presumably, NOX2 was up-regulated earlier than 1 day post-HS to produce high superoxide amounts [96]. High levels of superoxide have been exhibited to up-regulate the SOD expression [117,118]. It may be assumed that SOD up-regulation may lead to NOX2 down-regulation [119]. Corresponding to NOX5, NOX2 regulation may involve other factors apart from ROS. Reports demonstrated that HS-induced small ubiquitin-like modifier 1 (SUMO1) down-regulates ROS production through NOX2 [120]. This may clarify the negative NOX2 regulation upon HS.

In addition, SODs are ubiquitous enzymes catalyzing the superoxide anions’ dismutation to hydrogen peroxide. SOD1 (cytoplasmic isoform) and SOD3 (extracellular isoform) contain Cu and Zn, whereas SOD2, the mitochondrial isoform, has Mn in its reactive site [121]. In another study, Habashy et al. [110] noticed that the *SOD1* mRNA expression did not alter 1 day post-HS, but raised at 12 days post-HS. Particular functions between SODs may be the result of subcellular location [121]. Notwithstanding, it is obvious that birds’ HS exposure results to mRNA expression alterations in NADPH oxidases that induce SOD1 up-regulation, one of the pivotal enzymatic antioxidant defenses as regards cell damages by superoxide anions. As reported by Schafer and Buettner [122] superoxide dismutase up-regulation is one of the mechanisms cells employ to control potential cytotoxicity induced by stress. These results were in accordance with Azad et al.’s [123] results, which described that cytoplasmic Cu/Zn-SOD (SOD1) activity raised in the Pectoralis major muscle after chronic exposure of chickens to HS.

Differently, through the antioxidant enzyme catalase (CAT), H_2_O_2_ is converted to water and oxygen, and through GPX, H_2_O_2_ is converted to water in a reaction, oxidizing GSH to its disulfide form (GSSG). Glutathione is regenerated from GSSG by GR. Data concerning the regulation of CAT, GPX, NADPH, and GR in both acute and chronic HS is insufficient. The GPX, CAT, and NADPH genes were negatively regulated at 1 day post-HS, presumably as a result of H_2_O_2_ raise. It has been demonstrated that exhibition to ROS negatively regulated CAT expression through hypermethylation of a CpG island in CAT promoter [124,125]. Τhe high levels of cellular H_2_O_2_ due to HS may have caused the negative regulation of GPX and CAT. This may be true not only at gene level, since in case of GPX, post-translational modifications have been reported irrespective of changes at gene expression levels [126].

Niu et al. [127] showed that S-glutahionylation of human cystathionine β-synthase increased its activity to enhance the production of cysteine and afterwards GSH under oxidative stress conditions. Recently, Habashy et al. [128] exhibited that under HS, cysteine is mainly incorporated into chickens’ tissues compared to any other amino acid. Under HS, dietary methionine (a cysteine precursor) should be raised to increase the trans-sulfuration pathway flux in order to convert homocysteine to cysteine. In another study, Eriksson et al. [129] exhibited that methionine is critical in reduction systems and cells protection against oxidative stress through an NADPH-independent pathway. It was exhibited that hepatocytes can preserve cytosolic redox homeostasis by utilizing NAPDH or methionine. *Nrf2* gene was slightly negatively regulated at 1 day post-HS, but not at 12 days post-HS.

The induction of NADPH and GST by electrophiles and antioxidants has been shown to be mediated by the activation of Nrf2 in human [130]. It should be also noticed that GST was positively regulated at 1 day post-HS, which commonly believed to accelerate the conjugations between GSH and 4-hydroxynonenal (HNE), a byproduct of lipid peroxidation generated upon HS [131]. GST negative regulation 12 days post-HS may be a result of GSH depletion. Further, Nrf2 activates the antioxidant responsive elements which induce the transcription of diverse genes in the redox homeostasis machinery [132]. Presumably, the early transcriptional alteration in Nrf2 during HS is essential to trigger a cascade of events to reserve redox homeostasis [110].

Al-Zghoul et al. in their experiment on thermal manipulated (TM) chicks illustrated that levels of *SOD2, GPX2, NOX4,* and *CAT* expression were significantly lower in the TM groups in two chicken breeds (Hubbard and Cobb) [133]. This demonstrates that the reduction of *NOX4* expression in the TM groups alleviates oxidative stress by decreasing NOX-induced ROS. Additionally, AvUcp expression levels in the Hubbard and Cobb TM groups generally increased after exposure to acute heat stress (AHS) compared to controls. These results indicate that TM through raising *AvUcp* mRNA levels has a positive effect on ROS reduction. In relation with the above results, TM treatment has a role in decreasing heat-induced oxidative stress, the latter of which can be ascertained by the reduction and elevation of *NOX4* and *AvUcp* mRNA levels, respectively. Lastly, they showed that TM directs thermotolerance acquisition in broiler chicken and indicated considerable differences among breeds [133].

Moreover, the relative mRNAs expression of HSP (60, 70, and 90) in the heart of broiler chickens were significantly elevated after exposure to HS for 2 h and then declined rapidly with further exposure. In addition, the up-regulation of these stress proteins in heart act as important biomarkers and protective proteins at the start of HS [102]. A summary of selected studies in chicken reporting the effects of heat stress on various factors, HSP, and selenoproteins is shown in Table 2.

### 4.3. Antioxidant Supplements during Heat Stress and Avian Antioxidant Transcriptome Response

Several nutritional approaches, such as supplementation of diets with phytochemicals, have been used in attempts to attenuate the negative effects of HS [147]. Vitamin E (Vit E) is a fat-soluble vitamin with antioxidant properties [148] that regenerates damaged tissues [149,150] during oxidative stress by participating in the GPX pathway causing enhanced chicken performance [151]. 

Furthermore, Vit E and Se combined supplementation proved to be the most effective inhibitor of lipid peroxidation. Dietary Vit E and Se supplementation led to significant increases in SOD and CAT levels in breast muscle of heat-stressed broilers. Selenium alongside with Vit E had a synergistic effect on SOD, GPX, and CAT activity. However, Shahnawaz Kumbhar showed that enzyme activity of GPX remained depressed in case of dietary Vit E [134]. Furthermore, MDA content in breast meat decreased by both Se and Vit E supplementation under HS condition [141]. That was in agreement with other findings, who observed a significant increase in tissue *GPX1, GPX4,* and selenoprotein P mRNA levels when broilers were fed Se and Se+Vit E supplemented diets. However, no effect on these indices was recorded in broilers given the Vit E supplemented diet.

Selenium performs its biological functions mainly through selenoproteins, as Se is mainly incorporated in selenoproteins’ active sites [152]. Selenoproteins like GPX1, GPX4, and selenoprotein P play vital roles in a variety of biological processes by participating in the antioxidant defense system. Different Se forms including inorganic, such as sodium selenite, organic, such as Se-enriched yeast, and nanoselenium (Nano-Se) have been compared to choose the best source of Se for maximizing the poultry production and health in oxidative or thermoneutral rearing conditions [102].

Thus, Nano-Se supplementation has been proposed for reduction of HS effects in broilers. In a study on the effects of dietary Nano-Se supplementation at 0.6 and 1.2 mg/kg of diet on growth performance, serum biochemical parameters, immune response, antioxidant capacity, and jejunal morphology of 29-d-old male broilers subjected to HS at 37 ± 1 °C for 14 d, it has been shown that heat-stressed broilers had lower FI and BMG, but higher FCR than those kept in thermoneutral condition. According to Safdari-Rostamabad’s [137] study outcomes in broilers after 48 days of HS exposure, FCR of broilers improved due to dietary supplementation with 1.2 mg/kg Nano-Se. Furthermore, the 1.2 mg/kg Nano-Se supplementation had better results than the 0.6 mg/kg or the control treatment concerning FCR and BMG, proposing that heat-stressed broilers’ performance can be improved with a supplementation rate of 1.2 mg/kg. This might be associated to HSPs (and other chaperones) and to proteolytic enzymes.

Hu et al. [153], compared the effects of Nano-Se and sodium selenite on the growth performance of broilers, Se concentrations in liver, serum, and breast muscle, activity GPX in serum, and retention of Se in the whole body and in liver tissue. It was showed that both Se sources comparably increased feed efficiency, average daily gain, survival ratio, and serum GPX activity. Nevertheless, Nano-Se supplementation in broilers notably improved Se transfer to the body from intestinal lumen, Se concentration in tissues and in the serum and retention of Se in the whole body. Also, performance parameters were not affected by different Se supplementation sources (Nano-Se, Se-enriched yeast and sodium selenite) in the non-stressed or oxidative-stressed broilers. However, Nano-Se triggered the most noticeable impact in oxidative stressed broilers [154]. In parallel with the above outcomes, Safdari-Rostamabad et al. demonstrated that during broilers’ grower and starter phases, Nano-Se supplementation at levels of 0.6 or 1.2 mg/kg had no beneficial effect [137]. Also, Nano-Se supplementation did not alleviate the adverse effect of HS on pancreas [137]. On the other hand, supplementation with Se enriched probiotics, facilitated an induction of the endogenous antioxidant defense system. These observations indicate that an improved antioxidant status could greatly attenuate heat-stress-induced HSPs expression.

In another study, when broilers were supplemented with Se enriched prebiotics (SP), a significant downregulation was observed in the expression of the HSPs (60, 70, and 90) heat stress biomarkers in the breast muscles of each experimental group compared with the control group. The SP group had a profound effect on decreasing the *HSP70* mRNA levels in comparison to control [136].

## 5. Bibliometric Evaluation

**Figure 1:**
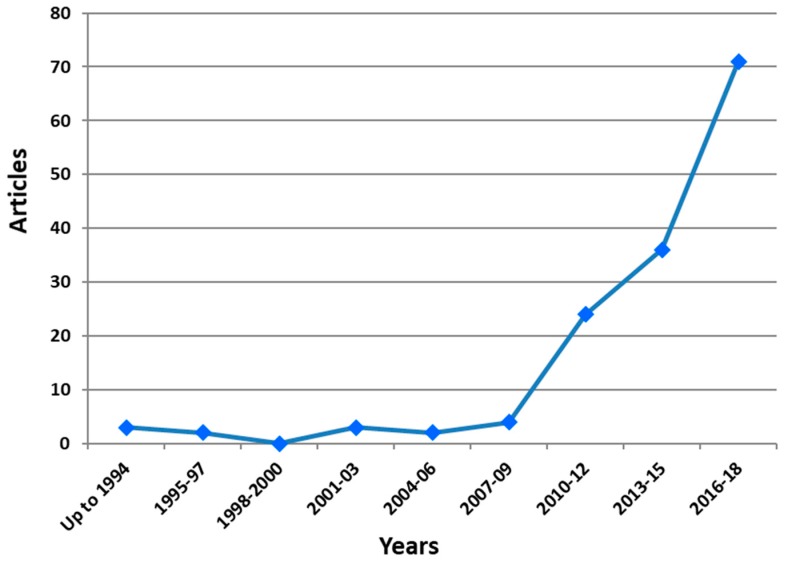
Temporal evolution of articles concerning selenoprotein transcriptome in chicken and its interactions with heavy metals.

The field of selenoprotein transcriptome in chicken and its interactions with heavy metals has recently been attracting more and more research interest (Figure 1). The search was performed using Scopus database and [TITLE-ABS-KEY((“selenium supplementation” OR selenium OR selenoproteins OR DIO1 OR “iodothyronine deiodinase 1” OR DIO2 OR “iodothyronine deiodinase 2” OR DIO3 OR “iodothyronine deiodinase 3” OR GPX1 OR “glutathione peroxidase 1” OR GPX2 OR “glutathione peroxidase 2” OR GPX3 OR “glutathione peroxidase 3” OR GPX4 OR “glutathione peroxidase 4” OR GPX6 OR “glutathione peroxidase 6” OR MRSB1 OR “methionine sulfoxide reductase B1” OR selenof OR “selenoprotein F” OR selenoh OR “Selenoprotein H” OR selenoi OR “selenoprotein I” OR selenok OR “selenoprotein K” OR selenom OR “selenoprotein M” OR selenon OR “selenoprotein N” OR selenoo OR “selenoprotein O” OR selenop1 OR “selenoprotein P1” OR selenop2 OR “selenoprotein P2” OR selenos OR “selenoprotein S” OR selenot OR “selenoprotein T” OR selenou OR “selenoprotein U” OR selenov OR “selenoprotein V” OR selenow OR “selenoprotein W” OR sephs2 OR “selenophosphate synthetase 2” OR TXNRD1 OR “thioredoxin reductase 1” OR TXNRD2 OR “thioredoxin reductase 2” OR TXNRD3 OR “thioredoxin reductase 3”) AND (Cd OR cadmium OR chromium OR Cr OR mercury OR Hg OR arsenic OR As OR lead OR Pb OR “heavy metals” OR “toxic elements”) AND (chicken OR chick OR chicks OR broiler OR hen OR egg) AND (mRNA OR transcriptome OR transcript OR RNA))] were used as keywords. Figure 1 shows the temporal evolution of the articles. As it is shown, there has been an exponential growth in the last 10 years. Related articles were more than 70 in the period 2016–2018 whereas in the period 2007–2009 were only 4. Authors anticipate that this trend will continue in the next years. 

**Figure 2:**
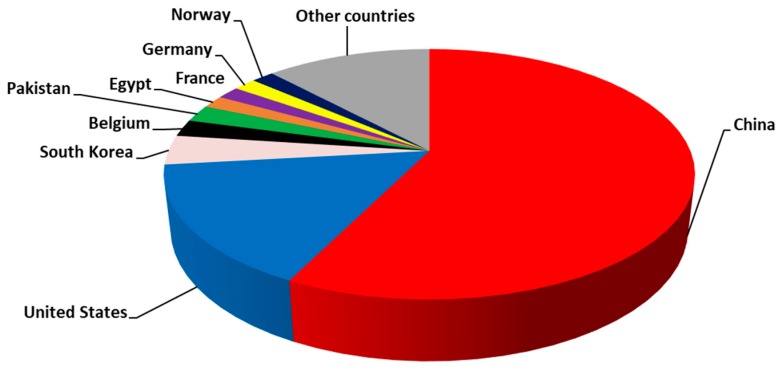
Articles per country, concerning selenoprotein transcriptome in chicken and its interactions with heavy metals.

Figure 2 shows the related research per country. China outnumbers all other countries, obtaining more than 50% of the total with 105 articles. As it is known, China is one of the greatest producers of plant protection products and fertilizers. These products could include heavy metals. Further, China has a high number of industries that could use or have as by-products heavy metals. Moreover, China’s GPD increases continuously and so more and more research is conducted. Another reason why most articles come from China is that the health issue of Keshan’s disease emerged, a congestive cardiomyopathy caused by a combination of dietary deficiency of Se and the presence of a mutated strain of Coxsackievirus. These symptoms were prevalent in a wide area extending from north-east to south-west China, all due to Se-deficient soil. The U.S. is in the second position with 29 articles, and South Korea, Belgium, Pakistan, and other countries follow. Articles are scattered in more than 60 journals. This fact could be explained due to the multidisciplinary character of the field. Although, “Biol. Trace Elem. Res.” dominates the field with 49 articles, while “Environ. Sci. Pollut. Res.” and “Poult. Sci.” are following with 6 articles each. These journals are highly cited, reflecting the importance of the field.

**Figure 3:**
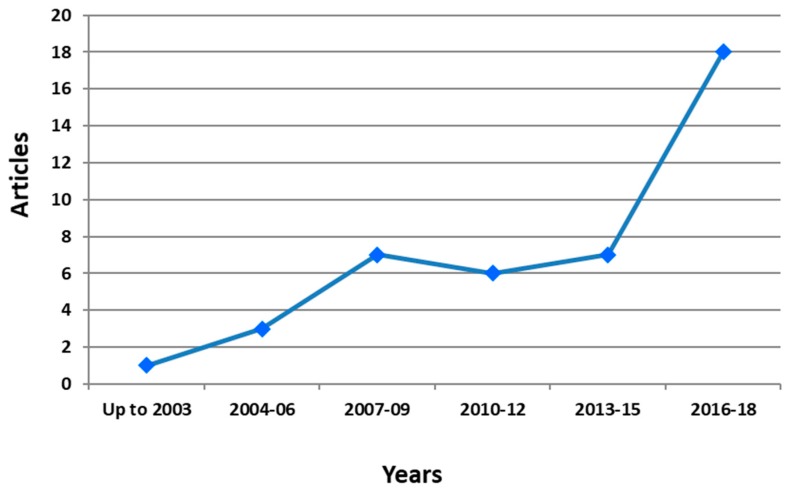
Temporal evolution of articles concerning selenoprotein transcriptome in chicken and its adaptation to heat stress and selenium deficiency.

Regarding research on selenoprotein transcriptome in chicken and its adaptation to HS and Se deficiency, the number of articles has also an increasing trend (Figure 3). The search was performed using Scopus database and TITLE-ABS-KEY[((“selenium supplementation” OR selenium OR selenoproteins OR DIO1 OR “iodothyronine deiodinase 1” OR DIO2 OR “iodothyronine deiodinase 2” OR DIO3 OR “iodothyronine deiodinase 3” OR GPX1 OR “glutathione peroxidase 1” OR GPX2 OR “glutathione peroxidase 2” OR GPX3 OR “glutathione peroxidase 3” OR GPX4 OR “glutathione peroxidase 4” OR GPX6 OR “glutathione peroxidase 6” OR MRSB1 OR “methionine sulfoxide reductase B1” OR selenof OR “selenoprotein F” OR selenoh OR “Selenoprotein H” OR selenoi OR “selenoprotein I” OR selenok OR “selenoprotein K” OR selenom OR “selenoprotein M” OR selenon OR “selenoprotein N” OR selenoo OR “selenoprotein O” OR selenop1 OR “selenoprotein P1” OR selenop2 OR “selenoprotein P2” OR selenos OR “selenoprotein S” OR selenot OR “selenoprotein T” OR selenou OR “selenoprotein U” OR selenov OR “selenoprotein V” OR selenow OR “selenoprotein W” OR sephs2 OR “selenophosphate synthetase 2” OR TXNRD1 OR “thioredoxin reductase 1” OR TXNRD2 OR “thioredoxin reductase 2” OR TXNRD3 OR “thioredoxin reductase 3”) AND (chicken OR chick OR chicks OR broiler OR hen OR egg) AND (“heat stress”))] were used as keywords. The number of related articles were more than doubled in last 3-year period (2016–2018). As depicted in Figure 3, 58 articles have been published until the end of 2018. Half of them were published after 2013. 

**Figure 4:**
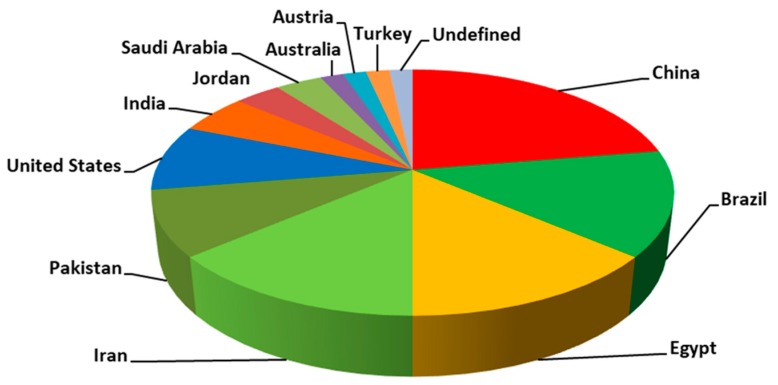
Articles per country, concerning selenoprotein transcriptome in chicken and its adaptation to heat stress and selenium deficiency.

Figure 4 includes the countries which have published relative articles. Asian countries dominate the field. Particularly, China is again in the first place with 13 articles. Brazil, Egypt, and Iran follow with 8 articles each, and Pakistan and United States with 5 articles each. Τhese countries are known for their warm climate and usually hot summers. Meanwhile, there is high demand for chicken meat in these countries. Therefore, the production of chicken is analogous. With regard to journals, “Biol. Trace Elem. Res.” leads with 11 articles.

## 6. Conclusions

It can be concluded that the toxic effects of Cd and Pb and the harmful effects caused by heat stress in chickens are alleviated via supplementation of Se. Studies highlight selenium’s role as antioxidant and modulator for the enzymatic and non-enzymatic antioxidant defense factors such as GSH, GPx, and TrxR in order to modulate the toxic effects of heavy metals in chicken. Further, Se alleviates the excess of mRNA expression levels of apoptotic factors, immune proteins, and heat stress proteins caused by heavy metals. In addition, over heat stress conditions, Se through upregulation of GPxs and other selenoproteins, eliminates heat stress proteins mRNA levels in many chicken tissues and reduces mRNA increase of inflammatory factors and other chicken immune responses.

## Figures and Tables

**Table 1:** A summary of selected studies in chicken describing effects of heavy metals on various factors, heat shock proteins (HSP), and selenoproteins.

Type of Supplementation	Heavy Metal	Tissue	Inflammation Factors and Other Proteins	Heat Stress Proteins	Cell Death Regulation Proteins	Selenoproteins	Other Results	Analytical Method	Reference
Na_2_SeO_3_ 2 mg/Κg	CdCl_2_ 150 mg/Kg	Liver (in vivo)	Se/Cd alleviation of increased mRNA levels of NF-κB, COX-2, PTGES, TNF-α, and IL-1 in relation to Cd treatment	Alleviation of increased mRNA/protein levels of HSP60, HSP70, HSP90 in relation to Cd treatment	No	No	Decrease of Cd induction (decrease of Li, B, Ca, Fe, Ti, Cu, Mo, Cd, Cr, Se, Sr, Ba, and Hg concentrations)	RT-PCR, Western blot	[47]
Na_2_O_3_Se 1 mg/Kg	Pb(CH3COO)_2_ 350 mg/Kg	Neutrophils (in vivo)	Decrease of (IL-1β, IL-1R, IL-4, IL-8, IL-10, IL-12, TGF-β4) increased the mRNA expression of IL-2 and IFN-γ	Decrease of protein HSP27, -40, -60, -70, -90 and mRNA of HSP60 and -70 in relation to Pb treatment	No	No	No	RT-PCR, Western blot	[35]
Na_2_SeO_3_ 2 mg/Kg	CdCl_2_ 150 mg/Kg	Pancreas (in vivo)	No	No	No	No	Se/Cd treatment alleviated the mRNA increase of T-SOD, CAT, GSH-Px, T-AOC caused by Cd toxicity in relation to control	ICP-MS, RT-PCR	[18]
Na_2_SeO_3_ 1 mg/Kg Se	Pb(CH_3_COO)_2_ 350 mg/L	Testes (in vivo)	No	No	Se/Pb: downregulation of caspase-3, caspase-12 in relation to Pb treatment	GPX upregulation in Se treatment and alleviation of GPX downregulation induced by Pb in Se/Pb treatment	No	RT-PCR	[66]
Na_2_SeO_3_ 2 mg/Kg	CdCl_2_ 218.44 mg/Kg	Ovary (in vivo)	Se/Cd treatment alleviated the mRNA increase of HK2, PK, SDH, PbHX, LC3, Atg5, Beclin 1, Dynein, Lc3-I, Lc3-ll, mTOR caused by Cd toxicity in relation to control	No	No	No	No	q-PCR, Western Blot	[49]
Na_2_SeO_3_ 1mg/Kg	Pb(CH_3_COO)_2_ 350 mg/L	Kidney (in vivo)	No	No	caspase-3, caspase-12, Bcl-2 increase in Pb group and alleviation of increase in Pb/Se group	No	No	RT-PCR, Western Blot	[69]
No	CdCl_2_ 10 mg/Kg	Spleen (in vivo)	AKT and mTOR decrease	HSP70 decrease	No	No	[Ca, Cr, Se, Sr, Sn, Ba decrease and Na, Mg, V, Fe, Mo, Cu, Zn, Cd increase] LC3-I, LC3-II, Beclin-1,NF-kB, p-JNK/JNK increased	ICP-MS, qRT-PCR, Western Blot	[48]
Na_2_SeO_3_ 2 mg/Kg	CdCl_2_ 150 mg/Kg	Kidney (in vivo)	Cd group: increase in mRNA levels of COX-2, NF-κB, PTGES, and TNF-α Se/Cd group: alleviation of mRNA level increase of NF-kB and TNF-α. COX-2 and PTGES were not influenced	No	No	(Decrease in the mRNA levels of GPX2, GPX3, DIO3, selenoprotein K, -N, -O, -Pb, -S, -T, -U, and -W between the Cd group and control, not in the mRNA levels of the GPX1, GPX4, DIO1, DIO2, Txnard1, -2, -3, selenoprotein H, -I, -M, Sep15, Sepp1, Sepx1, SPS2) AND (between Cd/Se group and control GPX2, GPX3, selenoprotein T, -U, and -W smaller decrease)	No	RT-PCR	[28]
Na_2_SeO_3_ 0.02 mg/L	Pb(CH_3_COO)_2_ 12 mg/L	Spleen (in vitro)	No	No	Se/Pb: increase due to Pb exposure of p53, Bak, caspace-3, caspase-9, Cyt-c and decrease of PL3K, Akt, Bcl2 alleviated via Se supplementation	No	Se alleviated the increase of MDA levels due to Pb and alleviated the decrease in antioxidant enzyme activity (GPX, SOD, and CAT) due to Pb Additionally, ROS levels in the control group and the Se group were not significantly different. Se alleviated the increase of ROS levels due to Pb	RT-PCR, Flow cytometry, Western Blot	[71]
Na_2_SeO_3_ 2 mg/Kg	CdCl_2_ 150 mg/Kg	Spleen (in vivo)	No	No	Se/Cd: caspase-3, caspase-9 small alleviation of mRNA increase due to Cd treatment but extensive alleviation of increase of caspase-3 protein levels	Se/Cd treatment alleviate the decrease of TrxR1, GPX1 due to Cd treatment	Cd increased H_2_O_2_ and MDA and SOD but T-AOC, CAT decreased Bax, Cyt-c, Bak alleviation of increase due to Cd	ICP-MS, Western Blot, RT-PCR	[54]
Na_2_SeO_3_ 2 mg/Kg	CdCl_2_ 150 mg/Kg	Liver (in vivo)	Se/Cd: iNOS alleviaton of increase of mRNA levels due to Cd similarly in protein levels	No	Se/Cd: caspase-3, caspase-9, p53 alleviation of increase of mRNA levels due to Cd protein levels	No	Se/Cd: Cyt-c alleviation of increase due to Cd	RT-PCR, Western Blot, TUNEL assay	[45]
Na_2_SeO_3_ 2 mg/Kg	CdCl_2_ 150 mg/Kg	Neutrophils (in vivo)	Se/Cd: alleviated increase of mRNA levels of COX-2 and the decrease of TNF-α due to Cd	Se/Cd: HSP 40, HSP 70, HSP 90 alleviation of increase of mRNA levels due to Cd but in HSP 60 is the same with Cd group	Se/Cd: NF-κB, IL-2, IL-4, IL-17, IFN-γ alleviation of mRNA levels increase due to Cd and IL-10, IL-1β, iNOS alleviation of decrease of mRNA levels due to Cd	No	No	RT-PCR	[41]
Na_2_SeO_3_ 1mg/Kg	Pb(CH_3_COO)_2_ 350 mg/Kg	Testes (in vivo)	No	Se/Pb: alleviation of increase of HSP27, -40, -60, -70, -90 mRNA levels caused by Pb toxicity in relation to control	No	DIO1, DIO2, DIO3, GPX1, GPX2, GPX3, GPX4, selenoprotein H, -I, -K, -M, -O, -Pb, -S, -T, -U, -W, -15, Sepn1, Sepp1, Sepx1, SPS2, Txnrd1, -2 and -3 increase of mRNA expression in Se group and alleviation of increase in Se/Cd group	No	qRT-PCR	[36]
Na_2_SeO_3_ 1mg/L	Pb(CH_3_COO)_2_ 350 mg/L	Neutrophils (in vivo)	Se treatment slightly increased TNF-α 3, Cox-2, iNOS, NF-κB mRNA levels in relation to control while Pb increased TNF-α3, Cox-2, iNOS, NF-κB mRNA levels in relation to control and Se/Pb treatment alleviated aforementioned increase of mRNA levels	No	GPX2, GPX3, GPX4, DIO1, DIO2, DIO3, Txnrd1, Txnrd2, Txnrd3, SPS2, Sepx1, Sepp1, selenoprotein S, -K, -O, -U, -H, -15, and -M, significantly higher in Se group than in control and slightly higher in Pb treatment in relation to control. Se/Pb treatment intensified the increase in Pb treatment in relation to control	No	No	RT-PCR, Western Blot	[70]
Na_2_SeO_3_ 0.02 mg/L	CdCl_2_ 0.2 mg/L	Neutrophils (in vitro)	Se/Cd treatment alleviated the increase of mRNA levels of IL-1β,IL-4, IL-10, IFN-γ, NF-κB, iNOS, COX-2, TNF-α, and PGE2 due to Cd present and also alleviated the mRNA levels decrease of IL-17 due to Cd toxicity in relation to control	No	No	No	No	TUNEL assay, RT-PCR, Western blot	[46]
Na_2_SeO_3_ 1mg/L	Pb(CH_3_COO)_2_ 350 mg/L	Testes (in vivo)	Se/Pb treatment alleviated the increase of NF-κB, TNF-α, COX-2, PTGE mRNA, and NF-κB protein levels due to Pb toxicity in relation to control	Se/Pb treatment alleviated the mRNA levels increase of HSP60, -70, -90 due to Pb toxicity in relation to control	No	No	Se/Pb treatment alleviated the 90 days Pb accumulation in testes	qRT-PCR, Western Blot	[67]
Na_2_SeO_3_ 1mg/L	Pb(CH_3_COO)_2_ 350 mg/L	Bursa of Fabricius (in vivo)	Se/Pb alleviated the mRNA increase of IL-2, IL-4, IL-6, IL-12β, IL-17, and the mRNA decrease of IFN-γ caused by Pb toxicity in relation to control	No	No	No	T-AOC, GPX, GST, SOD, and CAT activities increase in Se treatment in relation to control, in Pb treatment T-AOC, GPX, GST, SOD, and CAT activities decreased in relation to control and Se/Pb treatment alleviated this decrease	qRT-PCR	[65]
Na_2_SeO_3_ 1mg/L	Pb(CH_3_COO)_2_ 350 mg/L	Nervous Tissues (in vivo)	No	No	Se/Pb treatment alleviate the decrease of Bcl2 protein/ mRNA levels while alleviate the increase of protein/mRNA levels in p53, Bax, Cyt-c, caspases-3 due to Pb toxicity	No	No	qRT-PCR	[72]
Na_2_SeO_3_ 1 mg/L	Pb(CH_3_COO)_2_ 350 mg/L	Heart (in vivo)	Se/Pb treatment alleviated the increase of NF-kB, TNF-a, COX-2 and PTGEs mRNA levels due to Pb toxicity	No	No	Se/Pb treatment alleviated the decrease of mRNA levels of GPX1, -2, -3, and -4, Txnrd1, -2, -3, DIO1, -2, -3, selenoprotein N1, -K, -S, -T, -O, -H, -M, -15, -U, -Pb, Sepp1, Sepn1, Sepw1, Sepx1, SPS2 due to Pb toxicity in relation to control	No	qRT-PCR	[73]
Na_2_SeO_3_ 2 mg/Kg	CdCl_2_ 150 mg/Kg	Heart (in vivo)	No	No	Se/Cd treatment alleviated the increase of JNK, AMPK and PPARα due to Cd exposure and alleviated the decrease of P-JNK	No	No	qRT-PCR, Western Blot	[75]
Na_2_SeO_3_ 1 mg/L	Pb(CH_3_COO)_2_ 350 mg/L	Kidney (in vivo)	No	No	Se/Pb treatment alleviated the decrease of mRNA levels of mfn1, drp1, opa1, mff, mfn2 due to Pb toxicity	No	Se/Pb treatment alleviated the decrease of Cpx, SOD, MDA, ATPase activities, Mitochondrial complex V, -II, -I activities due to Pb toxicity	RT-PCR, Western Blot, TUNEL assay	[68]
Na_2_SeO_3_ 1 mg/L	Pb(CH_3_COO)_2_ 350 mg/L	Lymphocytes (in vivo)	Se/Pb treatment alleviated the mRNA increase of iNOS, TNF-a, COX-2, NF-KB due to Pb in relation to control	Se/Pb treatment alleviated the increase of mRNA levels of HSP27, -40, -60, -70, -90 due to Pb toxicity in relation to control	No	No	No	RT-PCR	[63]
Na_2_SeO_3_ 1 mg/L	Pb(CH_3_COO)_2_ 350 mg/L	Cartilage (in vivo)	No	No	No	Se alleviated the downtrend of the expression of GPX1, -2, -4, Txnrd2, Txnrd3, DIO1, DIO2, selenoprotein I, -U, Sepx1, selenoprotein K, -W, -O, -M, Sep15, Sepnn1, selenoprotein S, and -T induced by Pb in relation to control	Se/Pb treatment alleviated the concentration of Pb in sword cartilage tissue	qRT-PCR, ICP-MS	[74]
Na_2_SeO_3_ 1 mg/L	Pb(CH_3_COO)_2_ 350 mg/L	Liver (in vivo)	Se/Pb treatment alleviated the increase of mRNA levels of NF-κB, TNF-α, COX-2, PTGEs, and iNOS due to Pb toxicity in relation to control	Se/Pb treatment alleviate the increase of mRNA levels of HSP27, -40, -60, -70, -90 caused by Pb toxicity in relation to control	No	No	No	qRT-PCR	[31]
Na_2_SeO_3_ 0.02 mg/L	CdCl_2_ 0.2 mg/L	Splenic Lymphocytes (in vitro)	Se/Pb treatment alleviated the decrease of IL-1β, -2, -4, -10, -17, and IFN-γ mRNA levels due to Cd toxicity in relation to control	No	No	No	No	qRT-PCR	[44]
Na_2_SeO_3_ 0.02 mg/L	CdCl_2_ 0.2 mg/L	Lymphocytes (in vitro)	No	No	No	Se/Cd treatment alleviated the decrease of selenoprotein K, -N, -T, -S mRNA levels caused by Cd toxicity in relation to control	No	qRT-PCR	[51]
Na_2_SeO_3_ 10 mg/Kg	CdCl_2_ 150 mg/Kg	Immune organs (serum, thymus, spleen, Bursa of Fabricius) (in vivo)	Se/Pb treatment alleviated the increase of iNOS activity and NO production caused by Pb in relation to control	No	Se/Pb treatment alleviated the mRNA increase of p53 and apoptotic rates while alleviated the mRNA decrease of Bcl2 in relation to control	No	No	qRT-PCR, TUNEL assay	[42]
Na_2_SeO_3_ 10 mg/Kg	CdCl_2_ 150 mg/Kg	Cerebrum and Cerebellum (in vivo)	Se/Cd treatment alleviated the increase of iNOS mRNA/protein levels and NO activity induced by Cd toxicity in relation to control	No	No	Se/Cd treatment alleviated the GPX mRNA levels decrease caused by Cd toxicity in relation to control	Se/Cd treatment alleviated Pb accumulation	qRT-PCR, FAAS	[42]
Na_2_SeO_3_ 0.02 mg/L	CdCl_2_ 0.2 mg/L	Splenic Lymphocytes (in vitro)	No	No	Se/Cd treatment alleviated the mRNA increase of Bak, caspase-3, -9, p53 and Cyt-c and alleviated the mRNA decrease of Bcl-x, Bcl-2, CaM induced by Cd toxicity in relation to control	No	No	DCF, TUNEL Assay, qRT-PCR	[53]
Na_2_SeO_3_ 0.02 mg/L	CdCl_2_, 0.2 mg/L	Splenic Lymphocytes (in vitro)	No	Se/Cd treatment alleviated the mRNA levels increase of HSP27, -40, -60, -70, -90 induced by Cd toxicity in relation to control	No	No	No	qRT-PCR	[76]

**Table 2:** A summary of selected studies in chicken reporting effects of heat stress on various factors, HSP, and selenoproteins.

Type of Supplementation	Tissue	Selenoproteins	Heat Stress Proteins	Antioxidant Capacity	Other Results	Analytical Techniques/Methods	References
Na_2_SeO_3_ 0.2 mg/Kg, Vit E 250 mg	Breast muscles	Se/Vit E: upregulation of Gpx1, Gpx4 and selenoprotein P in relation to control and Se group	HSP60, -70, -90 small mRNA decrease in Se group, no differences in Se/Vit E group	Se/Vit E and Se group: increase in concentration of CAT, SOD, GSH-P and MDA especially in Se/Vit E group in relation to control		RT-PCR	[134]
BET 1g/Kg, Vit E 250 mg/Kg, Se 0.8 mg/Kg	Breast muscle			BET, Vit E and Se: increased GPx activity	BET reduced respiratory rate	GPx Assay	[135]
No	Liver	Gpx1 mRNA decrease		Heat stress treatment: increase NOX1, NOX3, DUOX2, GST, CAT, SOD1, GR, CASP6 mRNAs and decrease of CYBB, NOX4, NOX5, NADPH mRNAs		RT-PCR	[110]
Na_2_SeO_3_ 0.30 mg/Kg, Se-yeast 0.30 mg/Kg	Breast muscles	upregulation of Gpx1, Gpx4 in both Se treatments	Downregulation of HSP70 in inorganic Se group, Se-yeast group showed a further downregulation HSP70 mRNA levels compared to control and inorganic Se group		Improved organoleptic meat characteristics (meat drip loss, water holding capacity, and shear force)	qRT-PCR, HG-AFS	[136]
Νano-selenium 1.2 mg/Kg	Jejunal tissue				Decreased the plasma concentrations of LDL-C and AST, but linearly increased that of HDL-C before heat exposure. Moreover, the cholesterol concentration was lower in broilers fed diets supplemented with 0.6 mg/kg Nano-Se than that in the control ones. Heat stress decreased the plasma total protein concentration, but increased the AST activity	Enzymatic Kits	[137]
Vit A 16.000 IU/kg, Na_2_SeO_3_ 0.50 mg/kg					Se/Vit E group: no significant change in egg quality in relation to control but significant changes in hen performance		[138]
DL-α-tocopherole acetate 500 mg/Kg, Na_2_SeO_3_ 0.5 mg/Kg					Se/Vit E group: synergistic effect between Se and Vit E in alleviation of heat stress		[139]
Na_2_SeO_3_ 1.5 mg/Kg, PAMK 200 mg/Kg	Spleen		Alleviation of increase of mRNA expression of HSP90, GRP-78 caused by heat stress		Se/PAMK group: alleviation of increase of expression of Bcl-2, caspase-3, ATF4, ATF6, IRE due to heat stress	qRT-PCR, Western Blot	[106]
Organic Se 0.3mg/Kg, Cr 2 mg/Kg, Zn 40 mg/Kg	Blood			Improved performance and antioxidant responses (reduced LP and increased superoxide dismutase)			[140]
SeMet 1 mg/Kg α-tocopherol acetate 250 mg/Kg	Breast				Se/Vit E group: growth performance was not improved but improved lipid oxidation of breast meat	AAS, MDA determination	[141]
Se 3 mg/Kg, PAMK 200 mg/Kg	Bursa of Fabricius, spleen, thymus		Se/PAMK: Improved alleviation of mRNA increase of HSP60, -70, -90		Se/PAMK group: Alleviation of mRNA increase of TNF-a, IFN-γ, IL2 and IL4 caused by heat stress	qRT-PCR	[142]
Se 3 mg/Kg, PAMK 200 mg/Kg	Endoplasmic reticulum of Spleen tissue		Higher alleviation of HSP27 and -70 increase in Se/PAMK group			qRT-PCR, Western Blot Analysis	[108]
Se 1mg/Kg, Vit E 250 mg/Kg					Better immune responses	Enzymatic methods	[143]
Na_2_SeO_3_ 0.028 mg/Kg	Liver, Breast muscle			GSH-Px activity increase due to Se supplementation		Enzymatic methods	[144]
Vit E 250 mg/kg, Se 1 mg/kg	Pectoralis muscle			SOD and Gpx activity increase	There was not a significant interaction in broiler growth performance between dietary treatments and environmental temperature	Enzymatic methods	[145]
Se 0.3 g/Kg, TP 10 g/Kg	Blood				Se/TP significantly reduced plasma triglycerides no significant effects on plasma hormones T	Enzymatic Methods	[146]

## Abbreviations

ATF4	Activating transcription factor 4
AvUcp	Avian uncoupling protein
Bak	BCL2 antagonist/killer 1
Bcl-2	B-cell lymphoma 2 gene
Bcl-xL	B-cell lymphoma-extra large
BNIP3	Adenovirus E1B 19 kDa protein-interacting protein 3
CaM	Calmodulin protein gene
CHOP	CCAAT-enhancer-binding protein homologous protein
COX-2	Cyclooxygenase 2
DIO	Iodothyronine deiodinase
eIF2α	Eukaryotic Initiation Factor 2
GPX	Glutathione peroxidase
GRP78	Unfolded protein response regulator
GSH	Glutathione
GST	Glutathione S-transferases
ICAM-1	Intercellular Adhesion Molecule 1
IFN-γ	Interferon gamma
IL	Interleukin
iNOS	Inducible NO synthase
NF-κB	Nuclear factor kappa-light-chain-enhancer of activated B cells
NO	Nitric oxide
p53	Tumor protein p53 gene
PERK	Protein kinase RNA-like endoplasmic reticulum kinase
PGE2	Prostaglandin E2
PTGES	prostaglandin E synthase
SECIS	Selenocysteine insertion sequence
SEPHS2	Selenophosphate Synthetase 2 gene
SEPP1	Selenoprotein P
Sepx1	Methionine-R-sulfoxide reductase B1
SOD	Superoxide dismutase
TGF-β4	Transforming Growth Factor-β
TNF-α	Tumor necrosis factor alfa
TNR-α	Tumor necrosis receptor alfa
TXNRD	Thioredoxin reductase
